# Orthotopic transplantation of retinoblastoma cells into vitreous cavity of zebrafish for screening of anticancer drugs

**DOI:** 10.1186/1476-4598-12-71

**Published:** 2013-07-09

**Authors:** Dong Hyun Jo, Dain Son, Yirang Na, Manyoung Jang, Jae-Hoon Choi, Jin Hyoung Kim, Young Suk Yu, Seung Hyeok Seok, Jeong Hun Kim

**Affiliations:** 1 Fight against Angiogenesis-Related Blindness (FARB) Laboratory, Clinical Research Institute, Seoul National University Hospital, Seoul, Republic of Korea; 2 Tumor Microenvironment Research Center, Global Core Research Center, Seoul National University, Seoul, Republic of Korea; 3 Department of Biomedical Sciences, College of Medicine, Seoul National University, Seoul, Republic of Korea; 4 Department of Microbiology and Immunology and Institute of Endemic Disease, College of Medicine, Seoul National University, Seoul, Republic of Korea; 5 Department of Life Science, College of Natural Sciences, Hanyang University, Seoul, Republic of Korea; 6 Department of Ophthalmology, College of Medicine, Seoul National University, Seoul, Republic of Korea

**Keywords:** Anticancer drug screen, Orthotopic transplantation, Retinoblastoma, Zebrafish

## Abstract

**Background:**

With high throughput screening, novel therapeutic agents can be efficiently identified. Unfortunately, researchers only resort to in vitro cell viability assays for screening of anticancer drugs for retinoblastoma, the most common intraocular cancer in the childhood. Current available animal models of retinoblastoma require more than 2 weeks for tumour formation and the investigation of the efficacy of therapeutic agents. In this study, we established a novel orthotopic transplantation model of retinoblastoma in zebrafish as an in vivo animal model for screening of anticancer drugs.

**Methods:**

We injected retinoblastoma cells into the vitreous cavity of zebrafish at 48 hours after fertilization. Eyeballs of zebrafish were scanned daily under the confocal laser microscope, and the tumor population was quantitatively analyzed by measuring the mean intensity of green fluorescent protein (GFP). Transplanted retinoblastoma cells were isolated to perform further analyses including Western blotting and reverse transcriptase-polymerase chain reaction to confirm that retinoblastoma cells maintained their characteristics as tumor cells even after transplantation and further isolation. To figure out the potential of this model for screening of anticancer drugs, zebrafish were cultured in Ringer’s solution containing carboplatin and melphalan after the injection of retinoblastoma cells.

**Results:**

The degree of the tumor population was dependent on the number of retinoblastoma cells injected and maintained stably for at least 4 days. Transplanted retinoblastoma cells maintain their proliferative potential and characteristics as retinoblastoma cells after isolation. Interestingly, systemic application of carboplatin and melphalan demonstrated significant reduction in the tumor population, which could be quantitatively analyzed by the estimation of the mean intensity of GFP.

**Conclusions:**

This orthotopic retinoblastoma model in zebrafish is expected to be utilized for the screening of anticancer drugs for the treatment of retinoblastoma.

## Background

For the effective treatment of cancer, it is crucial to select proper regimens of anticancer drugs, which are based on the robust development of various regimens to improve efficacy and minimize toxicity. Last few decades are blossomed with the introduction of novel therapeutics such as targeted therapy or immunotherapy for cancers [1,2]. In the pediatric cancers, there are also many attempts for the introduction and actual uses of novel therapeutics [3]. Unfortunately, that was not the case in the treatment of retinoblastoma, the most common intraocular malignancy in childhood but an uncommon disease of the incidence of 1/20,000 births worldwide [4,5]. Currently, carboplatin-based regimens are widely utilized in the systemic chemotherapy and melphalan is commonly employed in the intraarterial chemotherapy, which addresses the tumor by the administration of anticancer drugs to ophthalmic artery via catheterization [6,7]. Although these approaches have yielded satisfactory clinical outcomes, there are still patients who are compelled to undergo enucleation, the complete removal of the eyeball, resulting in irreversible vision loss for the lifetime.

Novel therapeutic agents can enhance the efficacy of currently utilized administration modalities including intravenous, intraarterial, and intravitreal injection [8]. For the development and screening of novel therapeutic agents, the effective screening tools are desperately required. As for retinoblastoma, a previous attempt on multiple screening of anticancer drugs simply utilized in vitro cell viability assays and measurements of chemosensitivities in the human tumor clonogenic assay using primary retinoblastoma cells and established cell lines [9]. However, there is no effective in vivo animal model for multiple screening of anticancer drugs at one time. Currently available animal models including mice with genetic aberrations and murine orthotopic transplantation models require more than 2 weeks to form tumors; therefore, they are not suitable for rapid and high throughput screening of anticancer drugs [10,11].

In this study, we transplanted retinoblastoma cells into the vitreous cavity of zebrafish to establish a novel orthotopic transplantation model of retinoblastoma in zebrafish that can be utilized for high throughput screening of anticancer drugs. Zebrafish are suitable for extensive testing of multiple drugs because of relatively low maintenance cost, accessibility of in vivo imaging, and tiny size [12,13]. Especially, we investigated the potential of orthotopic transplantation of retinoblastoma to mimic the tumor microenvironment as much as possible. In addition, we can quantitatively analyze the degree of the tumor population in this model with a public image processing program. Furthermore, there is no change in the characteristics of tumor cells between before and after the injection, demonstrating the stability of transplanted cells as tumor cells during the study. Also, we identified the possibility of this model as a screening tool for various anticancer drugs with 2 widely utilized anticancer drugs for retinoblastoma, carboplatin and melphalan.

## Results

### Human retinoblastoma cells are injected into the vitreous cavity of zebrafish embryo at 48 hpf

**Figure 1:**
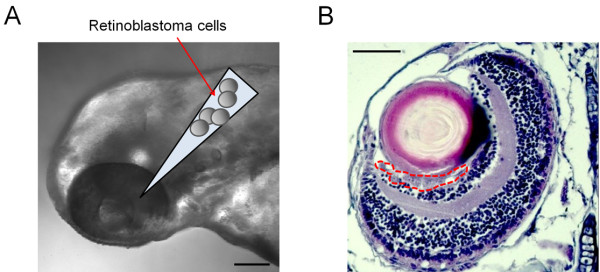
**Human retinoblastoma cells are injected into the vitreous cavity of zebrafish embryo at 48 hpf.** Via glass capillaries attached to the microinjector with the pneumatic pump system, retinoblastoma cells are injected into the vitreous cavity of zebrafish embryo **(A)**. 4- μm thin paraffin section demonstrates the existence of retinoblastoma cells 2 days after the injection in the vitreous cavity, the bowl-like hollow space between the lens and the retina **(B)**. Scale bar, 100 μm.

To establish the orthotopic transplantation model of retinoblastoma in zebrafish, we injected retinoblastoma cells into the vitreous cavity of zebrafish embryo at 48 hours post fertilization (hpf) (Figure 1A). Normally, the larval pigment pattern of zebrafish was developed completely within 6 days after fertilization [14]; therefore N-phenylthiourea (PTU) was used 1 day after fertilization to inhibit pigmentation of the eyeballs of zebrafish to maximize the visibility of the tumor population under the confocal laser microscope. PTU of the concentration utilized in this study (0.2 mM) did not show inhibitory effect on the proliferation of retinoblastoma cells by itself (data not shown). In zebrafish, the vitreous cavity is a bowl-like hollow space between the lens and the retina. When the cells were adequately injected into this space, they could spread along the vitreous cavity and maintain their population. As shown in Figure 1B, retinoblastoma cells showed stable maintenance in the vitreous cavity of zebrafish 2 days after the injection (days post injection, dpi).

### The degree of the tumor population is dependent on the number of retinoblastoma cells injected

**Figure 2:**
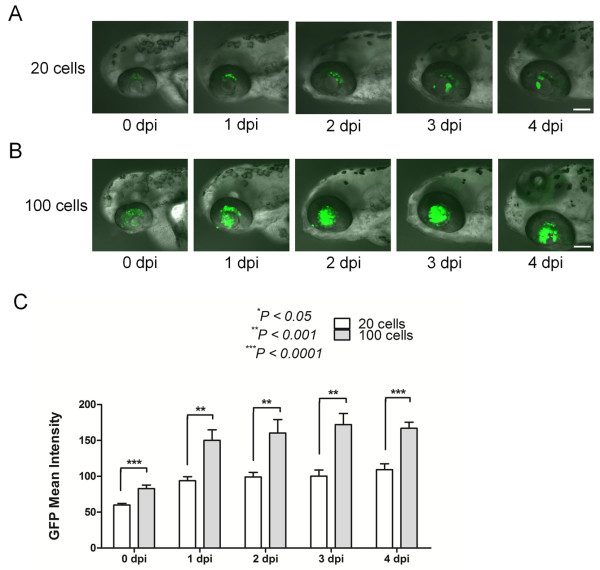
**The degree of the tumor population is dependent on the number of retinoblastoma cells injected.** We injected 20 and 100 retinoblastoma cells into the vitreous cavity of zebrafish, and the eyeballs of zebrafish embryos were scanned daily through the confocal laser microscope 20 cells, **(A**; 100 cells, **B)**. Scale bar, 100 μm. The tumor population was quantitatively analyzed as the mean intensity of GFP expression at 4 dpi with the public image processing program, ImageJ **(C)**. Data of 8 zebrafish per group from at least 2 independent experiments. dpi, day(s) post injection; GFP, green fluorescent protein.

To figure out the potential of the quantification of the data in this novel zebrafish model of retinoblastoma, we intravitreally injected retinoblastoma cells, SNUOT-Rb1 cells which we previously established [15], of different numbers, 20 and 100 cells (Figure 2A and B). To reduce variations between injections, we performed injection on over 200 zebrafish at a sitting with the pneumatic pump system. With the aid of microinjectors with glass capillaries and the regulator of injection pressure in the pneumatic pump system, we could minimize the intra-experiment and inter-experiment variations. For the quantification of the data, the mean intensity of green fluorescent protein (GFP) expression was measured using ImageJ software.

As expected, the degree of the tumor population was determined by differential amount of retinoblastoma cells injected (Figure 2C). The mean intensity of GFP expression was selected as the representative value for the tumor population instead of the total area of GFP expression in that the total area only represented the 2-dimensional distribution of tumor cells, not the 3-dimensional constitution of them. Interestingly, the degree of the tumor population was maintained from 1 dpi to 4 dpi (Figure 2C). The stable maintenance of the tumor population and the differential population according to the number of injected cells are 2 important characteristics that make up the potential of this method of orthotopic transplantation of retinoblastoma cells as an animal model of retinoblastoma.

### Transplanted retinoblastoma cells are successfully isolated and maintain their characteristics as tumor cells

**Figure 3:**
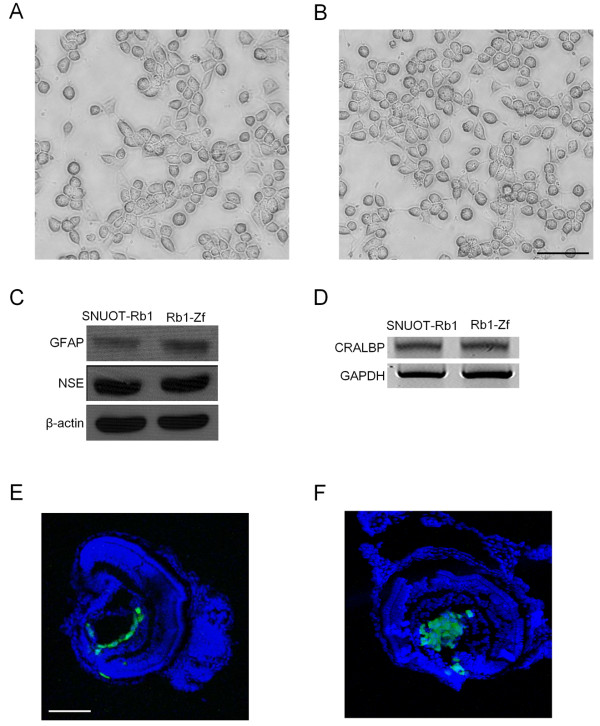
**Transplanted retinoblastoma cells are successfully isolated and maintain their characteristics as tumor cells.** Transplantation and further isolation of retinoblastoma cells do not induce morphological changes in retinoblastoma cells Before injection, **(A**; After transplantation and isolation, **B)**. Scale bar, 100 μm. Western blotting for glial differentiation marker, GFAP and neuronal differentiation marker, NSE demonstrates no definite change between preinjected and isolated retinoblastoma cells **(C)**. RT-PCR shows stable expression of CRALBP gene, a marker for retinal constituent cells **(D)**. As with the first injection of retinoblastoma cells, reinjection of isolated cells demonstrates the stable tumor population 2 days after the injection First injection, **(E**; Reinjection of isolated cells, **F)**. Scale bar, 100 μm. Rb1-Zf, isolated cells from zebrafish into which SNUOT-Rb1 cells were injected.

As shown in Figure 2C, the tumor population was maintained from 1 dpi to 4 dpi. This phenomenon may be due to the stability of this model system; however, it can be due to the alteration of the characteristics of tumor cells after the transplantation into the vitreous cavity. To verify that the transplanted cells retained their characteristics as retinoblastoma cells, we isolated retinoblastoma cells at 4 dpi from the zebrafish. Because we transduced GFP containing lentiviral particles into the cells before transplantation, we could identify transplanted cells by confirming the expression of GFP. Furthermore, the selection media containing puromycin was utilized throughout the isolation process to selectively isolate transplanted retinoblastoma cells. As expected, the morphology of retinoblastoma cells did not change with transplantation and further isolation (Figure 3A and B). To demonstrate the maintenance of previously reported biochemical characteristics of SNUOT-Rb1 cells after the transplantation and isolation [15], we performed Western blot for glial fibrillary acidic protein (GFAP, glial differentiation marker) and neuron-specific enolase (NSE, neuronal differentiation marker), demonstrating no change between SNUOT-Rb1 cells and isolated cells (Figure 3C). These patterns demonstrated that transplanted cells retained the characteristics of blastoma cells that shared glial and neuronal differentiation markers. The expression of cellular retinaldehyde-binding protein (CRALBP), the marker of retinal constituent cells, also exhibited no definite change between 2 types of cells (Figure 3D).

The potential of formation of the tumor population was examined by reinjecting isolated cells into the vitreous cavity of zebrafish. Similarly with the first injection (Figure 3E), reinjection of retinoblastoma cells effectively induced the tumor population (Figure 3F). Further cultivation of isolated cells showed that the cells retained unique characteristics of SNUOT-Rb1 cells of adherent and rapid growth.

### A novel orthotopic retinoblastoma model in zebrafish can be utilized for the screening of anticancer drugs

**Figure 4:**
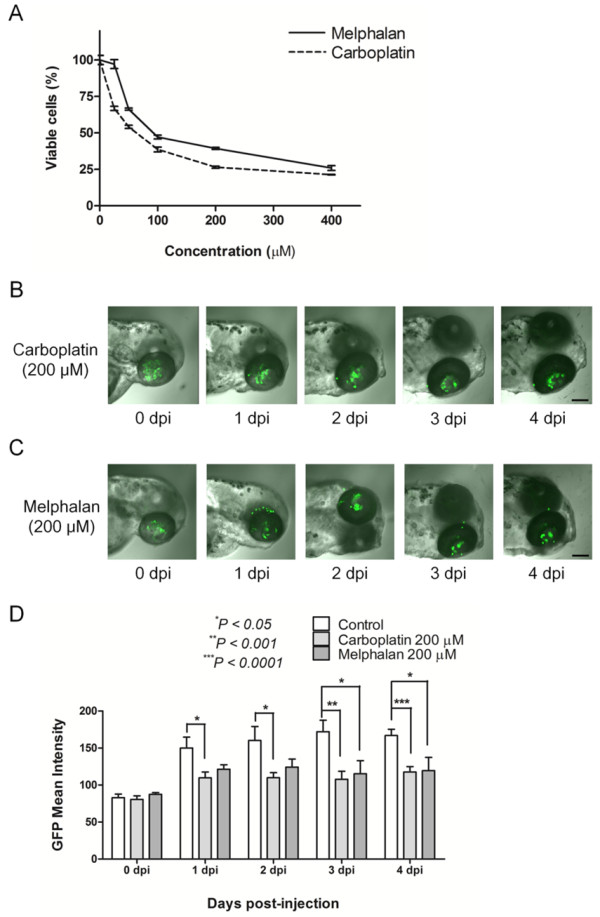
**A novel orthotopic retinoblastoma model in zebrafish can be utilized for screening of anticancer drugs.** Retinoblastoma cells are treated with carboplatin and melphalan of various concentrations from 25 to 400 μM **(A)**. Systemic application of both anticancer drugs diluted in fresh Ringer’s solution induces reduction of the tumor population in zebrafish Carboplatin treatment, **(B**; Melphalan treatment, **C)**. Scale bar, 100 μm. Quantitative analysis of the tumor response in this model yields stable results **(D)**. Data of 8 zebrafish per group from at least 2 independent experiments. dpi, day(s) post injection; GFP: green fluorescent protein.

We established this orthotopic retinoblastoma model in zebrafish with the purpose of utilizing for the screening of anticancer drugs. Carboplatin and melphalan were selected as representative drugs in this study, because carboplatin is one of the main anticancer drugs in the regimens for systemic chemotherapy [7], and melphalan is widely utilized in the regimen for intraarterial chemotherapy, which aims at the control of primary tumor or subretinal and vitreous seedings [16,17]. The treatment of retinoblastoma cells with carboplatin and melphalan for 48 hours induced reduction in the cell viability by 74% and 61% at the concentration of 200 μM, respectively (Figure 4A). As we observe in the clinical settings, these therapeutic agents showed inhibitory effect on proliferation of SNUOT-Rb1 cells.

Similarly with in vitro antiproliferative effect, 2 anticancer drugs demonstrated to reduce the tumor population in zebrafish (Figure 4B and C). To demonstrate anticancer activity of both drugs, zebrafish were cultured in Ringer’s solution containing 200 μM anticancer drugs right after the injection of 100 retinoblastoma cells. As shown in Figure 4B and C, therapeutic agents seemed to induce dispersion of retinoblastoma cells, not joining as a tumor in Figure 2B. We also compared the mean intensity of GFP expression between the control and the treatment groups. As expected, carboplatin and melphalan reduced the expression of GFP at 4 dpi by 30% (P < 0.0001) and 28% (P = 0.0312), respectively, compared to the control (Figure 4D). Like in these examples of carboplatin and melphalan, anticancer drugs can be screened in this novel orthotopic transplantation model for retinoblastoma in zebrafish.

## Discussion

The development of novel effective regimens is one of the best parts in the treatment of cancer. In addition, the investigation into proper administration options is required in localized tumors such as retinoblastoma, which originates from the inner layers of the retina and extends into the adjacent structures such as vitreous cavity, optic nerve, and choroid [18,19]. In this regard, researchers on the treatment of retinoblastoma have struggled to find novel therapeutic agents and modalities to deliver anticancer drugs to tumors. Particularly, current studies are mainly focused on the development and validation of novel therapeutic modalities such as intraarterial and intravitreal chemotherapy plus conventional intravenous chemotherapy [8]. With these efforts, tumors of group A to C according to the International Classification of Retinoblastoma (ICR) demonstrate the treatment success rate of over 90% by intravenous chemotherapy with carboplatin, vincristine, and etoposide [6]; intraarterial chemotherapy with melphalan can be utilized as salvage treatment after chemoreduction failure or primary treatment for advanced stages, group D or E according to the ICR [8]. However, studies on the novel therapeutic agents for retinoblastoma slightly lag behind compared to rapid increase in the development and introduction of targeted therapy or immunotherapy in other pediatric cancers [3]. Still, enucleation is the last choice for treatment failure with chemotherapy of various administration modalities; therefore, we speculated that the introduction of novel anticancer drugs might improve the clinical outcomes of current available modalities, minimizing the risk of irreversible vision and eyeball loss.

For the development of novel therapeutic agents, multiple screening of various candidate drugs is required. Once several candidate drugs are selected from in vitro experiments, adequate in vivo studies should be performed for identification of better drugs in animal models. However, current animal models of retinoblastoma are not suitable for high throughput screening of drugs in that tumor is formed after more than 2 weeks and we cannot perform experiments on many mice at one time. For example, the orthotopic transplantation model in mice requires 4 to 8 weeks to demonstrate the efficacy of anticancer drugs, although the tumor formation is evident in all the pups [10]. Similarly, researchers wait 2 weeks after the birth to form tumors in a genetic mouse model of retinoblastoma (Chx10-Cre;Rb(lox/lox);p107(−/−);p53(lox/lox)) [11].

In this study, we completed 1 round of experiments within 1 week after the fertilization and performed injection of retinoblastoma cells into the vitreous cavity on more than 200 zebrafish at a sitting. Transplantation of tumor cells to zebrafish has been utilized for investigation of tumor biology [20,21]. In addition, a few attempts explored the possibility of zebrafish models as tools for screening of anticancer drugs [13,22,23]. In these models, tumor cells from oral squamous cell carcinoma and leukemia cell lines are injected through yolk sac. We attempted the different approach from these studies in that we injected tumor cells into the vitreous cavity, with which retinoblastoma interacts in real patients. As shown in Figure 1, we successfully injected tumor cells into the vitreous cavity of zebrafish embryos at 48 hpf.

Interestingly, the highest increase in the GFP expression occurred between 0 dpi and 1 dpi and the level of GFP expression maintained stably throughout the study period, when we quantitatively analyzed the data. We speculated the presumptive reasons of this phenomenon as follows: 1) right after the injection, tumor cells might be inevitably packed. The emission of fluorophores might be interfered by other obscuring tumor cells or intraocular structures such as the lens in front of tumor cells when we observed the cells under the confocal laser microscope. 2) In this regard, after the redistribution of tumor cells from 0 dpi to 1 dpi, the expression of GFP was increased. The more important part with the alterations of GFP expression is that there was stable expression of GFP between 1 dpi and 4 dpi. In the normal culture condition, SNUOT-Rb1 cells double their numbers every 24 hours [14]. However, in the vitreous cavity of zebrafish and previous murine models of mice, doubling of tumor cells is not that fast [10]. The interaction between the tumor and the vitreous might be different from that between the tumor and the culture media.

The distribution patterns as well as the mean intensity of GFP expression were different according to the number of injected cells. We can observe dispersed cells when we injected only 20 cells (Figure 2A). In contrast, the injection of 100 retinoblastoma cells induced more ordered distribution of tumor cells as shown in Figures 2B and 3E. So we decided to inject 100 cells for further experiments on investigating into the potential of this model for screening of anticancer drugs.

Interestingly, we found the potential of this model as a tool for multiple screening of anticancer drugs with carboplatin and melphalan. We necessarily selected these 2 currently utilized drugs because they are widely utilized in real clinical settings for retinoblastoma patients. Interestingly, both drugs demonstrated effective anticancer activity on this model. These results might provide the possibility of this model as a screening tool for the evaluation of anticancer drugs which are in the development process. Furthermore, as previously mentioned, we can inject more than 200 zebrafish embryos at a sitting; therefore, more than 20 different candidate drugs can be screened with 1 session of experiments. Quantitative analysis of the tumor population yielded stable and reproducible results, demonstrating the usefulness of this model. After the image was captured with the confocal laser microscope, the quantitative data can be easily obtained with the public image processing program, ImageJ, by a few clicks.

## Conclusions

In this model, we performed orthotopic transplantation of retinoblastoma cells into the vitreous cavity of zebrafish. In this novel animal model of retinoblastoma, it was possible to quantitatively analyze the tumor population and the response of tumors to anticancer drugs with a public image processing program. Transplanted retinoblastoma cells retained their characteristics as tumor cells during the study period. We speculated that this novel orthotopic transplantation model for retinoblastoma in zebrafish can be a gateway for the development of anticancer drugs for retinoblastoma, one of the tumors that lag behind in the view of the development and introduction of novel therapeutic agents.

## Methods

### Zebrafish

Adult wild-type zebrafish, purchased from a local aquarium farm and maintained in the laboratory facility, were utilized for producing embryos by breeding. Zebrafish were raised at 28.5°C in alternate dark–light cycles of 13 and 11 hours, respectively. Twenty-four hours after fertilization, zebrafish embryos were placed in fresh Ringer’s solution with 0.2 mM PTU (Sigma-Aldrich, St. Louis, MO) to inhibit pigmentation of eyeballs. Care, use, and treatment of animals were done in agreement with the Association for Research in Vision and Ophthalmology for the use of animals in ophthalmic and vision research and the guidelines established by the Seoul National University Institutional Animal Care and Use Committee.

### Cell culture

SNUOT-Rb1 cells, from the previously established retinoblastoma cell line by our group [15], were maintained in RPMI 1640 medium (WelGENE, Daegu, Korea) supplemented with 10% fetal bovine serum (FBS; Gibco BRL, Rockville, MD) and 1% penicillin-streptomycin solution (Invitrogen, Carlsbad, CA) at 37°C in the humidified atmosphere of 95% air and 5% CO_2_. The cell line underwent the authentication by DNA fingerprinting analysis with short tandem repeat markers by Korean Cell Line Bank on November 22, 2012. For the visualization of cells with GFP, SNUOT-Rb1 cells were transfected with cop GFP Control Lentiviral Particles (sc-108084; Santa Cruz Biotechnology, Santa Cruz, CA) according to the manufacturer’s instructions. To select stable clones expressing GFP, cells were maintained in the culture media containing 4 μg/ml puromycindihydrochloride (sc-108071; Santa Cruz Biotechnology) for 2 weeks.

### Intravitreal injection of retinoblastoma cells

At 48 hpf, zebrafish embryos were anesthetized with 0.042 mg/ml Tricaine (ethyl 3-aminobenzoate methanesulfonate; Sigma-Aldrich). Eight to 10 hours before the injection, zebrafish embryos were dechorionated if necessary. For the stable injection, zebrafish were placed on the 1.7% agarose gel containing 1 ppm methylene blue. Then, using the Pneumatic PicoPump (PV820; World Precision Instruments, Sarasota, FL), cells of the indicated number were injected into the vitreous cavity of zebrafish embryos via glass capillaries attached to the Hamilton syringe (Hamilton Company, Reno, NV) under the stereomicroscope (Leica S6 E; Leica Microsystems, Wetzlar, Germany). Eyes of zebrafish were scanned daily on the Coverglass-Bottom dish (SPL Life Sciences, Pocheon, Republic of Korea) by the confocal laser microscope (Fluoview FV1000; Olympus, Tokyo, Japan) to record the alterations in the tumor population.

### Quantification of tumor population

We obtained the images of the eyeballs of zebrafish 1 hour, 24, 48, 72, 96 hours after the injection of retinoblastoma cells with the confocal laser microscope (Fluoview FV1000, Olympus). The mean intensity of GFP expression was measured using the ImageJ program (1.46r; National Institutes of Health, Bethesda, MD) [24]. After opening the image file captured with the confocal laser microscopy in the program, the color threshold was set using the menu Image>Adjust>Color Threshold. Then, the area over the designated threshold (brightness 48) was selected automatically with the yellow demarcation line or by putting the ‘select’ button on the pop-up. The mean intensity of GFP expression was calculated by the menu Analyze>Tools>Color Histogram or putting the ‘RGB’ button twice on the pop-up in the menu Analyze>Histogram to demonstrate green histogram of the image (Different approaches were required to get the values from version to version). The values were demonstrated as ‘Mean’ or ‘gMean’ on the pop-up. Eight zebrafish were included per group for experiments.

### Isolation of retinoblastoma cells

Four days after the injection of retinoblastoma cells, about 150 zebrafish were collected in 4°C phosphate-buffered saline supplemented with 1% penicillin-streptomycin solution (Invitrogen). Then, the zebrafish were digested with 1.5 mg/ml collagenase from *Clostridium histolyticum* (Sigma-Aldrich) in Hank’s balanced salt solution supplemented with 5% FBS (Gibco BRL) and 1% penicillin-streptomycin solution (Invitrogen) at 37°C for 30 minutes. The isolated cells were suspended and incubated in RPMI 1640 (WelGENE) supplemented with 10% FBS (Gibco BRL), 1% penicillin-streptomycin solution (Invitrogen), and 4 μg/ml puromycindihydrochloride (Santa Cruz Biotechnology). After the sufficient colonies were formed, we proceeded on further analyses.

### Immunoblot

The cells were lysed with RIPA buffer containing a protease inhibitor (Roche, Penzberg, Germany). The lysates were centrifuged at 13,000 rpm at 4°C for 20 minutes. Then, the supernatants were delivered to new micro test tubes for further processes. Equal amounts of extracted proteins from the cells were separated by electrophoresis on 7.5% SDS-PAGE and transferred to nitrocellulose membranes (AmershamHybond ECL, GE Healthcare Bio-Sciences, Piscataway, NJ). The membranes were incubated with anti-GFAP antibody (1:1,000, ab53554, Abcam, Cambridge, United Kingdom), anti-NSE antibody (1:1,000, #9536, Cell Signaling Technology, Beverly, MA), and anti-β-actin antibody (1:3,000, A2066, Sigma-Aldrich) at 4°C overnight. Then, the membranes were incubated with species-specific horseradish peroxidase-conjugated secondary antibodies (Pierce, Thermo Scientific, Waltham, MA). After the treatment of membranes with Amersham ECL™ western blotting detection reagent (GE Healthcare Bio-Sciences), the membranes were exposed to the film (AmershamHyperfilm ECL, GE Healthcare Bio-Sciences).

### Reverse transcriptase-polymerase chain reaction (PCR)

Total RNA was extracted from the cells using TRIzol (Invitrogen) according to the manufacturer’s instructions. For the synthesis of cDNA, 1 μg of total RNA was mixed and reverse transcribed with oligo(dT)_15_ primer, Superscript II reverse transcriptase (Invitrogen Corp.) and dNTPs. Polymerase chain reaction (PCR) was performed with the resultant cDNA, 10X PCR buffer, 2.5 mMdNTPs, 10 mM forward and reverse primers, DNA polymerase (Corebiosystem, Seoul, Republic of Korea), and RNAse-free water. The primers for cellular retinaldehyde-binding protein (CRALBP) were 5′-TGGCAAAGTCAAGAAATCACC-3′ (forward) and 5′-CGTGGACAAAGACCCTCTCA-3′ (reverse) [25], and the resultant product was 313 bp. PCR was performed with denaturation in 5 minutes at 94°C, followed by 35 cycles of 30 seconds of denaturation at 94°C, 30 seconds of annealing at 60°C, and 30 seconds of elongation at 72°C. The primers for GAPDH were 5′-ACCACAGTCCATGCCATCAC-3′ (forward) and 5′-TCCACCACCCTGTTGCTGTA-3′ (reverse), and the resultant product was 500 bp. PCR was performed with denaturation in 5 minutes at 94°C, followed by 30 cycles of 30 seconds of denaturation at 94°C, 30 seconds of annealing at 65°C, and 30 seconds of elongation at 72°C. The PCR products were electrophoresed on 1% agarose gels containing ethidium bromide in a constant 100 V field.

### Preparation and treatment of anticancer drugs

Carboplatin (C2538) and melphalan (M2011) were purchased from Sigma-Aldrich. Zebrafish were cultured in fresh Ringer’s solution containing 200 μM anticancer drugs after the intravitreal injection of retinoblastoma cells. The solutions were changed every 24 hours. Eyes of zebrafishembyos were scanned daily on the Coverglass-Bottom dish (SPL Life Sciences) by the confocal laser microscope (Fluoview FV1000, Olympus).

### Cell viability assay

Cell viability was evaluated with 2-(4-Iodophenyl)-3-(4-nitrophenyl)-5-(2,4-disulfophenyl)-2H-tetrazolium (water soluble tetrazolium salt, WST-1) assay using EZ-Cytox Cell Viability Assay kit (Itsbio, Seoul, Republic of Korea) according to the manufacturer’s instruction. Briefly, SNUOT-Rb1 cells were plated in 96 well plates and cultured overnight (1 × 10^4^ cells per well). The cells were treated with anticancer drugs of different concentrations (25, 50, 100, 200, 400 μM) for 48 hours. Then, the reagent from EZ-Cytox Cell Viability Assay kit was applied to each well. After 2 hours of additional incubation, 96 well plates were shaken thoroughly on the shaker for 1 minute. Absorbance was measured at 450 nm using the microplate reader (VersaMax, Molecular Devices, Sunnyvale, CA). To confirm the results of WST-1 assay, direct estimation of viable cells using Trypan Blue Stain (Life Technologies, Carsbad, CA) was performed after the treatment with anticancer drugs of different concentrations for 48 hours.

### Statistical analysis

Differences of the values between experiments were assessed with the Student’s t-test. All statistical analyses were performed using GraphPad Prism (GraphPad Software, La Jolla, CA). The mean value and the standard error of the mean were shown in figures. P-values less than 0.05 were considered as statistically significant, and *, **, *** were designated as P < 0.05, P < 0.001, P < 0.0001, respectively, in figures.

## Abbreviations

CRALBP: Cellular retinaldehyde-binding protein; dpi: Day(s) post injection; FBS: Fetal bovine serum; GFAP: Glial fibrillary acidic protein; GFP: Green fluorescent protein; hpf: Hours post fertilization; ICR: International classification of retinoblastoma; NSE: Neuron-specific enolase; PCR: Polymerase chain reaction; PTU: N-phenylthiourea.

## Competing interests

The authors declare that they have no competing interests.

## Authors’ contributions

JHK and SHS designed the research; DHJ and DS performed the research; YN, MJ, JC, JHK and YSY contributed materials and established methods; DHJ and DS collected data; GHJ, DS, JHK and SHS analyzed data; and DHJ, DS, JHK and SHS wrote the paper. All authors read and approved the final manuscript.
